# Evaluation of emodepside in laboratory models of human intestinal nematode and schistosome infections

**DOI:** 10.1186/s13071-019-3476-x

**Published:** 2019-05-14

**Authors:** Tanja Karpstein, Valérian Pasche, Cécile Häberli, Ivan Scandale, Anna Neodo, Jennifer Keiser

**Affiliations:** 1 Department of Medical Parasitology and Infection Biology, Swiss Tropical and Public Health Institute, Basel, Switzerland; 2 University of Basel, Basel, Switzerland; 3 Drugs for Neglected Disease initiative, Chemin Louis-Dunant 15, 1202 Geneva, Switzerland

**Keywords:** Emodepside, Drug repurposing, Soil-transmitted helminthiases (STH), Hookworms, *Trichuris* spp., Nematodes, Trematodes, *Schistosoma* spp.

## Abstract

**Background:**

Helminthiases are very prevalent worldwide, yet their treatment and control rely on a handful of drugs. Emodepside, a marketed broad-spectrum veterinary anthelminthic with a unique mechanism of action, undergoing development for onchocerciasis is an interesting anthelmintic drug candidate. We tested the *in vitro* and *in vivo* activity of emodepside on nematode species that serve as models for human soil-transmitted helminth infection as well as on schistosomes.

**Methods:**

*In vitro* viability assays were performed over a time course of 72 hours for *Trichuris muris*, *Necator americanus*, *Ancylostoma ceylanicum*, *Heligmosomoides polygyrus*, *Strongyloides ratti*, *Schistosoma mansoni* and *Schistosoma haematobium*. The drug effect was determined by the survival rate for the larvae and by phenotypical scores for the adult worms. Additionally, mice infected with *T. muris* and hamsters harboring hookworm infection (*N. americanus* or *A. ceylanicum*) were administered orally with emodepside at doses ranging from 1.25 to 75 mg/kg. Expelled worms in the feces were counted until 3 days post-drug intake and worms residing in the intestines were collected and counted after dissection.

**Results:**

After 24 hours, emodepside was very active *in vitro* against both larval and adult stages of the nematodes *T. muris*, *A. ceylanicum*, *N. americanus*, *H. polygyrus* and *S. ratti* (IC_50_ < 4 µM). The good *in vitro* activity was confirmed *in vivo*. Hamsters infected with the hookworms were cured when administered orally with 2.5 mg/kg of the drug. Emodepside was also highly active *in vivo* against *T. muris* (ED_50_ = 1.2 mg/kg). Emodepside was moderately active on schistosomula *in vitro* (IC_50_ < 8 µM) 24 h post-drug incubation and its activity on adult *S. mansoni* and *S. haematobium* was low (IC_50_: 30–50 µM).

**Conclusions:**

Emodepside is highly active against a broad range of nematode species both *in vitro* and *in vivo*. The development of emodepside for treating soil-transmitted helminth infections should be pursued.

**Electronic supplementary material:**

The online version of this article (10.1186/s13071-019-3476-x) contains supplementary material, which is available to authorized users.

## Background

Helminths affect a fifth of the world population and their associated morbidities include general fatigue, food malabsorption or iron deficiency anemia [1–4]. They are an important public health issue in low and middle income countries, where they enhance the vicious cycle of poverty notably by reducing school attendance and productivity [5, 6]. The most prevalent helminthiases are schistosomiasis (primarily caused by *Schistosoma haematobium*, *S. japonicum* and *S. mansoni*) that affects more than 250 million people and soil-transmitted helminthiases (STH) that account for more than 1.5 billion infected cases worldwide [2, 4, 6, 7]. Infections with the hookworms *Ancylostoma duodenale* and *Necator americanus*, the whipworm *Trichuris trichiura*, the roundworm *Ascaris lumbricoides* and the threadworm *Strongyloides stercoralis* are grouped as soil-transmitted helminths, based on their mode of transmission [8, 9].

Preventive chemotherapy is the strategy of choice, recommended by the World Health Organization (WHO) to control these helminth infections. Schistosomiasis control relies on praziquantel while albendazole, mebendazole, levamisole and pyrantel pamoate are used against STH [7, 10, 11]. A recent meta-analysis showed that all four drugs used against STH have a limited and even decreasing efficacy against the parasites [12]. Also, the recent epidemiological survey from Crellen et al. [13] reported that the efficacy of praziquantel against *S. mansoni* was reduced, likely because of frequent mass drug administration campaigns (MDA). Together with the rising risk of drug resistance due to an intense use of the same drugs and the lack of lead molecules in the development pipeline, the discovery of new anthelmintic treatments is urgent [12, 14, 15].

As the expected return on investment for helminthiases is negligible, drug repurposing represents a sustainable approach and an effective strategy to expand the pool of active molecules, in particular when using veterinary anthelmintics as starting point [16]. Emodepside, is a broad-spectrum veterinary anthelmintic licensed under the name of Profender® and Procox® and is used in combination with praziquantel and toltrazuril, respectively [17]. Its activity has been demonstrated against a wide range of nematodes in the veterinary field [18–25]. Repurposing emodepside for human use started more than ten years ago with pre-clinical studies against filarial nematodes which may be considered surrogates of human filarial infections [26]. These promising results triggered in 2016 a phase I study with emodepside in healthy volunteers, which was then completed by single and multiple ascending dose studies [17, 27].

**Fig. 1:**
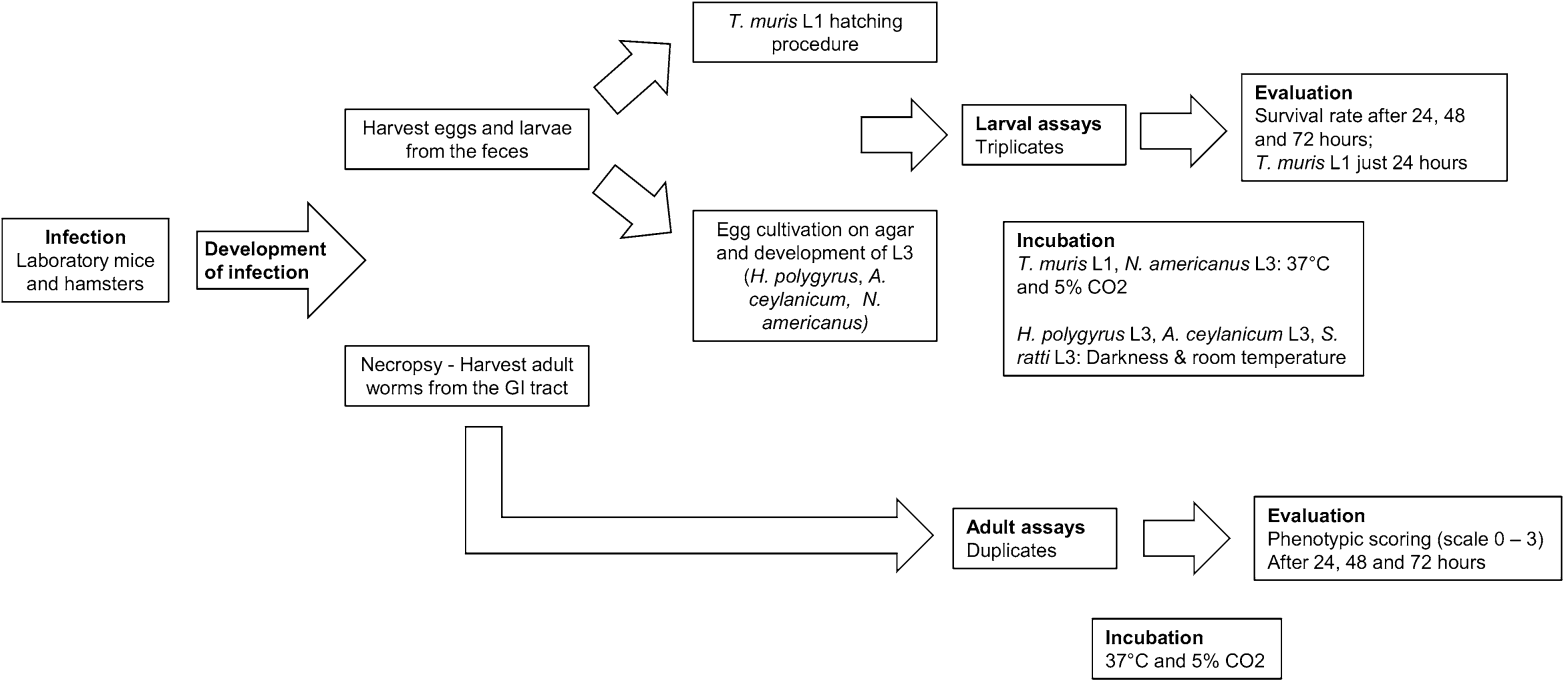
Workflow for the nematode assays

Aiming at possibly expanding its range of application in human medicine and in order to broaden previous work on laboratory models, we thoroughly tested emodepside against seven species of helminths *in vitro* and *in vivo* [28–30]. The drug was tested first *in vitro* on both larval and adult stages of *T. muris*, *A. ceylanicum*, *N. americanus*, *S. ratti* and *H. polygyrus*, as well as on *S. mansoni* and *S. haematobium*. The activity of emodepside was next tested *in vivo* in animal models infected *with T. muris*, *A. ceylanicum* and *N. americanus.* The experimental flow for the study on nematodes is presented in Fig. 1.

## Methods

### Drugs

Emodepside was purified by preparative high-performance liquid chromatography (HPLC) from the commercially available topical solution for cats Profender® (Bayer, Leverkusen, Germany). Profender® was diluted 1:1 (v/v) with acetonitrile (ACN), filtered (0.22 µm) and injected (10 mL) into a preparative HPLC (Varian ProStar system). The purification was performed on a ReproSil® 100 C18, 7 µm, 250 × 40 mm column (Dr. Maisch GmbH, Ammerbuch-Entringen, Germany) with double distilled water, 0.1% Trifluoroacetic acid (TFA) as solvent A and HPLC grade ACN (Buchs, Sigma-Aldrich) as solvent B at 20 ml/min flowrate. The following linear gradient was used: 0 min 20% B; 0.5 min 20% B; 34 min 100% B; 40 min 100% B. Fractions containing emodepside were then combined and lyophilized.

Emodepside’s ^1^H- and ^13^C-NMR spectra were recorded on Bruker 400 and 500 spectrometers and referenced to residual solvent peaks. LC-MS spectra of the purified compound were measured on an Acquity™ (Waters system) coupled to an Esquire HCT from Bruker (Bremen, Germany) using an Acquity UPLC BEH C18 column (2.1 × 50 mm, 1.7 μm) at 0.6 ml/min flow with a linear gradient of A (double distilled water 0.1% v/v formic acid) and B (ACN 0.1% v/v formic acid); t = 0 min, 5% B; t = 0.25 min, 5% B; t = 1.5 min, 100% B; t = 2.5 min, 100% B. NMR and MS spectra corresponded to that previously reported for emodepside (Segment solid-phase total synthesis of the anthelmintic cyclooctadepsipeptides PF1022A and emodepside) [31].

Levamisole was purchased in powder from Sigma-Aldrich (Buchs, Switzerland). Stock solutions of emodepside and levamisole (10 mM) were dissolved in pure dimethyl sulfoxide (DMSO, Sigma-Aldrich, Buchs, Switzerland) and stored until use at − 20 °C.

### Culture media

RPMI 1640 (Gibco, Waltham MA, USA) medium supplemented with 5% amphotericin B (250 µg/ml, Sigma-Aldrich, Buchs, Switzerland) and 1% penicillin 10,000 U/ml, and streptomycin 10 mg/ml solution (Sigma-Aldrich, Buchs, Switzerland) was used for the assays with *T. muris* adults and stage 1 larvae (L1), *H. polygyrus* adults and supplemented additionally with 10% inactivated fetal calf serum (iFCS; Bioconcept AG, Allschwil, Switzerland) for *T. muris* L1 hatching medium. RPMI 1640 supplemented with 5% amphotericin B (250 µg/ml), 1% penicillin (10,000 U/ml) and streptomycin (10 mg/ml) solution and 1% of the antibiotics mixture developed by Mäser et al. [32] was used for the *H. polygyrus* third stage larval (L3) assays. Adult *S. mansoni*, *S. haematobium* and *S. ratti* were incubated in RPMI 1640 medium supplemented with 5% iFCS and 1% penicillin (10,000 U/ml) and streptomycin (10 mg/ml) solution. Phosphate-buffered saline (PBS, Sigma-Aldrich, Buchs, Switzerland) supplemented with 1% penicillin (10,000 U/ml) and streptomycin (10 mg/ml) solution was used to incubate *S. ratti* L3 and to wash the adult worms. For the *S. mansoni* newly transformed schistosomula (NTS) assays, M199 medium (Gibco, Waltham MA, USA) supplemented with 5% iFCS and a mixture of antibiotics was used for the incubation [33]. *Ancylostoma ceylanicum* and *N. americanus* L3 stages were incubated in Hanksʼ balanced salt solution (HBSS; Gibco, Waltham MA, USA) supplemented with 10% amphotericin B and 1% penicillin (10,000 U/ml) and streptomycin (10 mg/ml) solution. The adult hookworms were kept in HBSS supplemented with 10% iFCS, 5% amphotericin B (250 µg/ml) and 1% penicillin (10,000 U/ml) and streptomycin (10 mg/ml) solution.

### Laboratory animals

Before the infection, all animals were left one week for acclimation in our facility. Three-week-old male Syrian golden hamsters (Charles River, Sulzfeld, Germany) were orally infected with 150 L3 of *A. ceylanicum* or subcutaneously with 150 *N. americanus* L3. Four-week-old female NMRI mice (Charles Rivers, Sulzfeld, Germany) were used for *S. mansoni* infection and injected subcutaneously with 100 cercariae. The same mice strain was used for growing *H. polygyrus* which were administered orally with 88 L3. Six-week-old female C57BL/6NRj mice (Janvier labs, Le Genest-Saint-Isle, France) were orally infected with 200 embryonated *T. muris* eggs. Three-week-old male Wistar rats (Janvier labs, Le Genest-Saint-Isle, France), were infected subcutaneously with 1300 *S. ratti* L3. Three-week-old male LVG Syrian Golden hamsters (Charles River, USA) were infected with *S. haematobium* cercariae at the Biomedical Research Institute (Atlanta, USA) before being sent to the Swiss Tropical and Public Health Institute.

All animals were kept in polycarbonate cages under environmentally-controlled conditions (temperature: 25 °C, humidity: 70%, 12:12 h light/dark photocycle) and had free access to tap water and rodent food. To guarantee a sustainable infection, dexamethasone (Sigma-Aldrich, Buchs, Switzerland) was supplied in the drinking water until 2 days before treatment for the NMRI mice infected with *H. polygyrus* (0.25 mg/l dexamethasone), the C57BL/6NRj mice infected with *T. muris* (1 mg/l dexamethasone) and the hamsters infected with the hookworms (0.5 mg/l dexamethasone). Animals were killed using the CO_2_ method to collect the adult worms for the in vitro studies as described below.

### Drug assays

To determine the half maximal inhibitory concentration (IC_50_) of the drug, each compound and concentration was tested in triplicates in the larval assays and in duplicates in adult worm assays. Parasites incubated in wells containing culture medium and DMSO corresponding to the highest drug concentration, served as negative controls and were included in every *in vitro* assay.

*Heligmosomoides polygyrus, S. ratti* and *A. ceylanicum* L3 larvae were then kept in the dark and at room temperature for 72 h. *Strongyloides ratti* L3 plates were sealed with Parafilm (Faust AG, Schaffhausen, Switzerland) before incubation. *T. muris* L1, *N. americanus* L3, *S. mansoni* NTS and the adult assays of all seven species were kept in the incubator for 72 h at 37 °C and 5% CO_2_.

#### In vitro tests on *N. americanus* and *A. ceylanicum* L3

*Necator americanus* and *A. ceylanicum* L3 were obtained from infected hamsters by the cultivation of eggs, gained by repeated filtration and centrifugation of the infected feces. The eggs were washed with tap water and cultivated on agar plates protected from light for 8 to 10 days at room temperature. The L3 were then kept in tap water supplemented with 5% amphotericin B (250 µg/ml), 1% penicillin (10,000 U/ml) and streptomycin (10 mg/ml) solution and used within 3 weeks. In each well of a 96-well plate (Sarstedt, Nümbrecht, Germany), 30 L3 were exposed to 4 serial dilutions (1:4) ranging from 0.016 µM up to 1 µM emodepside concentrations in a final volume of 200 µl.

#### In vitro tests on adult *N. americanus* and *A. ceylanicum*

Five weeks to six weeks post-infection (p.i.), the worms were collected directly from the hamster’s intestines. In a 24-well plate (Sarstedt, Nümbrecht, Germany), 3 to 4 adult worms per well were incubated in 2.5 ml drug solution with 4 different concentrations ranging from 0.005 µM to 0.5 µM for *A. ceylanicum* and with concentrations of 0.01 µM, 0.1 µM and 1 µM for *N. americanus*.

#### In vitro tests on *T. muris* L1

Six weeks after the infection of the mice, *T. muris* eggs were gained by filtration of their feces and storage in tap water for three months in the dark. To obtain the first stage larvae, egg hatching was triggered by incubation with *Escherichia coli* (BL21 strain) in hatching medium for 3 to 4 h at 37 °C in a wet chamber. For the assay, the L1 suspended in a total volume of 100 µl medium were placed in each well of a 96-well plate containing 14 emodepside concentrations ranging from 0.098 µM to 100 µM. Wells that contained levamisole (25 µM or 100 µM) served as positive control. The assays were kept in the incubator for 24 hours.

#### In vitro tests on adult *T. muris*

*Trichuris muris* adult worms were collected manually from the intestines of infected mice, 41 days p.i. The drug assays were performed in 24-well plates. In each well, 2 to 3 adult worms were incubated with the drug (1:4 serial dilutions ranging from 0.039 µM to 10 µM) in a final volume of 2.5 ml.

#### In vitro tests on *H. polygyrus* L3

The eggs were collected 2 weeks p.i. from mice feces and placed on agar at room temperature for 8 to 10 days in the dark. Forty L3 were exposed to emodepside at 3 different concentrations (0.625 µM, 2.5 µM and 10 µM) in a final volume of 100 µl.

#### In vitro assay on adult *H. polygyrus*

*Heligmosomoides polygyrus* adults were collected 2 weeks p.i. when dissecting mice intestines. In each well of 24-well plates 3 to 4 adult worms were exposed to emodepside (1:2 serial dilutions ranging from 0.125 µM to 1 µM) in 2.5 ml culture medium.

#### In vitro studies on *S. mansoni* NTS

*Schistosoma mansoni* (Liberian strain) cercariae were harvested from infected *Biomphalaria glabrata* snails and were then transformed into NTS [33, 34]. The drug assays and phenotypic screening were performed as described previously [33].

#### In vitro studies on adult *S. mansoni* and *S. haematobium*

Seven weeks p.i. the worms were extracted from the rodent mesenteric veins. Three single flukes or 2 pairs were placed in a final volume of 2.4 ml in each well of a 24-well plate exposed to 1:3 serial dilutions ranging from 3.7 µM up to 33.33 µM emodepside.

#### In vitro studies on *S. ratti* L3

The L3 were obtained following the procedure described by Garcia (1998) [35]. Thirty L3 were exposed to emodepside (1:4 serial dilutions ranging from 0.039 µM to 10 µM) in a final volume of 100 µl.

#### In vitro studies on adult S. ratti

The infected rats were dissected 3 weeks p.i. The intestines of the rats were excised, opened and immerged in PBS supplemented with penicillin/streptomycin. They were incubated for 4 h at 37 °C and 5% CO_2_ in order to detach the nematodes from the intestinal wall. A maximum of 15 worms were then transferred into the wells of 24-well plates and placed in 2 ml culture medium containing emodepside at 6 different concentrations ranging from 50 µM to 0.25 µM.

### Evaluation of the assays

The drug effect was evaluated 24, 48 and 72 h post-exposure. For evaluating the L3 assays, the larvae were stimulated if necessary by the addition of 50 µl (*T. muris* L1) to 100 µl (others spp.) hot water (≈ 80 °C) and the percentage of survival was determined by the ratio of moving larvae to the total number of larvae present in the well. The *N. americanus* L3 assay was an exception as the wells were stimulated by vigorous up and down pipetting. The adult worms of each parasite species were scored microscopically based on their phenotype, using a viability scale ranging from 3 to 0 (3: good motility and no morphological changes; 2: low motility and light changes in morphology; 1: very low motility and morphologically impaired; and 0: death). In case the adult worms did not move enough for a clear scoring, they were stimulated with hot water at the last evaluation time-point.

#### *Trichuris muris* in vivo studies

C57BL/6NRj mice were *orally* infected with 200 embryonated *T. muris* eggs. At 42 days p.i., the feces were collected and soaked for 1 hour in 0.9% sodium chloride, before the filtered suspension was examined under the microscope to determine the success of the infection. According to their infection intensities, the mice were equally assigned to the different groups. The compound was dissolved in 70:30 Tween 80-ethanol in ultrapure water (10% v/v) and was administered by gavage first at a dose of 75 mg/kg based on results from a previous study [28] followed by lower dosages from 1.25 to 10 mg/kg. Untreated mice served as controls. Until 3 days after drug administration the feces of the mice were examined for expelled worms. Six to seven days post-treatment, the animals were killed, their intestines were dissected, and the adult worms were collected and counted.

#### *Ancylostoma ceylanicum* and *N. americanus* in vivo studies

Hamsters were orally infected with 150 *A. ceylanicum* L3 or subcutaneously with 150 *N. americanus* L3. A fecal sample was collected from each hamster, just before treatment. The fecal samples were processed using an in-house sedimentation method to determine the infection intensity of each animal [36]. The different dosage and control groups were composed of hamsters evenly distributed depending on their infection status. *A. ceylanicum* infected hamsters were then treated on day 28 p.i. with a single oral dose of 2.5 mg/kg emodepside and *N. americanus* infected hamsters with a single oral dose of 1.25–10 mg/kg emodepside. Expelled worms were counted from each hamster from the collected feces until 72 h after treatment. One week post-treatment, the hamsters were euthanized and the worms remaining in their intestines were collected and counted.

### Data analysis

For the *in vitro* drug sensitivity assays, all viability scores and larval survival counts were averaged across replicates and normalized to the control wells. The effect of emodepside was determined by normalizing the mean parasite survival rate to the control wells. Based on these values, the IC_50_ values were calculated using CompuSyn software (ComboSyn Inc., version 1.0), as well as the *r*-values (the linear correlation coefficient) that reflects the goodness of the fit. For each assay, a minimal *r* value and viability of the controls was required. The detailed selection criteria are presented in Table 1.

**Table 1:** Mean IC_50_ values *in vitro* of emodepside tested on larval and adult stages of different helminths

Species	Replicates	No. of parasites per well^a^	24 hours	48 hours	72 hours
			Mean IC_50_ ± SD (µM)	Mean IC_50_ ± SD (µM)	Mean IC_50_ ± SD (µM)
*T. muris* (L1)	9	20–40	3.73 ± 6.54	–	–
*T. muris* (adults)	2	2–3	0.28 ± 0.15	0.043 ± 0.0089	0.022 ± 0.013
*H. polygyrus* (L3)	2	30	0.78 ± 0.086	0.9 ± 0.034	0.48 ± 0.05
*H. polygyrus* (adults)	3	3–4	0.57 ± 0.42	0.21 ± 0.13	0.25 ± 0.16
*A. ceylanicum* (L3)	2	30	0.14 ± 0.041	0.086 ± 0.08	0.25 ± 0.051
*A. ceylanicum* (adults)	3	2–3	0.0044 ± 0.0021	0.0015 ± 0.00078	0.0024 ± 0.002
*N. americanus* (L3)	2	30	0.77 ± 0.52	0.15 ± 0.069	0.083 ± 0.033
*N. americanus* (adults)	2	2–3	0.0031 ± 0.0011	0.0029 ± 0.0018	0.0021 ± 0.0012
*S. ratti* (L3)	4	30	0.73 ± 0.5	0.27 ± 0.21	0.25 ± 0.14
*S. ratti* (adults)	3	5–15	0.75 ± 0.57	0.21 ± 0.29	0.36 ± 0.32
*S. mansoni* (NTS)	2	100	7.79 ± 1.57	6.92 ± 0.21	2.48 ± 0.78
*S. mansoni* (adults)	2	2–3	50.4 ± 3.32	37.27 ± 10.47	34.1 ± 9.18
*S. haematobium* (adults)	2	2–3	40.51 ± 24.96	40.25 ± 6.49	36.73 ± 6.49

^a^Each assay included 2 to 3 wells per concentration/condition

*Notes*: The inclusion criteria used in our analysis were different for each stage and parasite. Minimal survival rates (larvae) or viability scores (adults and NTS) and IC_50_ r-values considered acceptable were as follows: *T. muris* L1 (survival rate: 60%; R = 0.7), adults (score: 2.5; R = 0.8); *H. polygyrus* L3 (70%; 0.9), adults (1.9; 0.8); *A. ceylanicum* L3 (55%; 0.7), adults (2; 0.7); *N. americanus* L3 (60%; 0.75), adults (2; 0.8); *S. ratti* L3 (60%; 0.75), adults (2; 0.7); *S. mansoni* NTS (2; 0.75), adults (1.5; 0.85); *S. haematobium* adults (2; 0.7)

*Abbreviation*: SD, standard deviation

To assess the effect of the drug *in vivo*, the mean numbers of living worms recovered in treated animals were compared to the controls. The worm burden reduction (WBR) was calculated as described previously [16]. The worm expulsion rate (WER) was determined by the number of dead worms excreted in the feces during 72 h after treatment, over the total number of worms (alive and dead) recovered during the necropsy. The Kruskal-Wallis test (Statsdirect version 3.1.20) were used to determine statistical significance of WBR at a level of 0.05. The median effective dose (ED_50_) values were calculated using CompuSyn software (ComboSyn Inc., version 1.0).

## Results

### In vitro studies

Table 1 and Additional files 1 and 2 summarize the mean IC_50_ values for each helminth species over 3 days post-drug exposure, except for *T. muris* L1 that were assessed after a 24 h incubation period. Emodepside showed IC_50_ values below 1 µM, within 24 h for all nematode species with the exception of *T. muris* L1 that had an IC_50_ of 3.7 µM. The highest drug activity was observed for adult hookworms. After one day of incubation, emodepside was highly active against adult *A. ceylanicum* and *N. americanus* with IC_50_ values below 0.005 µM, which were reduced by half over the incubation period (IC_50_ < 0.0025 µM). Decreasing IC_50_ values were also recorded over the 72 h incubation period for the adult worms of every species tested. Against *T. muris* adults emodepside showed an IC_50_ value below 0.3 µM after 24 h of drug exposure. This value decreased below 0.05 µM after another day of incubation. IC_50_ values in the range of 0.2 µM to 0.8 µM were observed for adult *H. polygyrus* and *S. ratti.*

IC_50_ values for all nematode L3 ranged from 0.9 µM to 0.08 µM. The IC_50_ values of *S. ratti* and *N. americanus* L3 decreased, while they decreased and increased over the 3 days incubation period for *H. polygyrus* and *A. ceylanicum* larvae, respectively. For the schistosomes *S. mansoni* and *S. haematobium*, IC_50_ values above 30 µM were calculated for adult worms while decreasing IC_50_ from 7.8 µM after 24 h to 2.5 µM after 72 h were observed for *S. mansoni* NTS.

For all the nematode species, the IC_50_ values were higher for the larval stages than for the adult worms. *Strongyloides ratti* was the only species where the difference between the two life-stages was less than 2-fold. The IC_50_ on adult worms was of about twice as high than for the larval stage for *H. polygyrus*, 13 times for *T. muris* and between 30 to 250 times for the hookworms *N. americanus* and *A. ceylanicum*. The exact opposite was observed for *S. mansoni*. At the 24 h and 48 h evaluation time-points, the IC_50_ values on NTS were 5 to 7 times lower than the ones measured on adult *S. mansoni*. When assessed after 3 days, a 13-fold difference was observed between *S. mansoni* NTS and the adult worms.

Emodepside was lethal (100% effect) *in vitro* on *A. ceylanicum* adults, *S. ratti* L3 and both life stages of *N. americanus*. Drug effects above 90% were observed at a concentration of 25 µM emodepside for *T. muris* L1 and at a concentration of 2.5 µM for adult worms. This was also the case for *A. ceylanicum* when incubated at 1 µM (L3) or 0.1 µM (adults) and *N. americanus* at 0.25 µM (L3) or 0.1 µM (adults). Effects of more than 75% were reached at 2.5 µM for *H. polygyrus* L3 and at 0.5 µM for adult worms. Emodepside had an effect above 75% at a concentration of 2.5  µM on *S. ratti* (L3) and showed a similar effect at a 10 times lower concentration when tested on adult worms.

### *In vivo* studies

The worm expulsion rates and worm burden reductions obtained with single-dose, oral emodepside against *T. muris* are summarized in Table 2. A high dose of 75 mg/kg emodepside resulted in complete elimination of all worms. At doses of 10 mg/kg and 2.5 mg/kg worm burden reductions of 85.9% and 69.6% and worm expulsion rates of 62.0% and 60.9%, respectively were observed. The lowest dose tested (1.25 mg/kg) showed low activity, with a worm burden reduction of 73.9% and worm expulsion rate of 5.3%. The worm burden reductions obtained with emodepside (all doses *versus* controls) were statistically significant (*t* = 7.18, *P* = 0.0073). Based on worm burden reductions we calculated an ED_50_ value of 1.2 mg/kg.

**Table 2:** *In vivo* dose response relationships of emodepside on *A. ceylanicum*, *N. americanus* and *T. muris*

	Dose (mg/kg)	Mean no. of worms ± SD	Worm expulsion rate (%)	Worm burden reduction (%)	*P*-value	ED_50_ (mg/kg)
*T. muris*						
Emodepside	75^c^	0	100	100	0.007^a^	1.2
	10^c^	18.8 ± 20.8	62.0	85.9		
	2.5^c^	36.5 ± 30.8	60.9	69.6		
	1.25^d^	133 ± 27.9	5.3	73.9		
Control 1^c^		120.8 ± 12.0	0	–		
Control 2^d^		121.7 ± 4.7	0	–		
*A. ceylanicum*						
Emodepside	2.5	0	100	100	0.014^a^	
Control		21.3 ± 2.6	0.6	–		
*N. americanus*						
Emodepside	10^c^	0	100	100	0.060^a^	0.5
	5^c^	0	100	100		
	2.5^d^	0.25 ± 0.5	87.5	93.8		
	1.25^d^	2.25 ± 2.3	40.0	43.8		
Control 1^c^		5.5 ± 6.1	5.6	–		
Control 2^d^		4.0 ± 1.4	0	–		

^a^Kruskal-Wallis test was applied to determine statistical significance on worm burden reduction of all doses *versus* controls

^c^Control 1 was used for this dose

^d^Control 2 was used for this dose

Single doses of 10 mg/kg and 5 mg/kg cured all *N. americanus*-infected hamsters. A worm burden reduction of 93.8% and a worm expulsion rate of 87.5% were observed at a dose of 2.5 mg/kg. Moderate activity (worm burden reduction of 43.8% and worm expulsion rate of 40.0%) was observed in *N. americanus*-infected hamsters with the lowest dose tested of 1.25 mg/kg (all doses, *t* = 3.52, *P* = 0.06). An ED_50_ value of 0.5 mg/kg was determined for emodepside in *N. americanus*-infected hamsters. To confirm that emodepside also acts on *Ancylostoma* spp. the minimum effective dose on *N. americanus* of 2.5 mg/kg was tested in the *A. ceylanicum* hamster model, which resulted in cure of all animals (*t* = 6.05; *P* = 0.014).

## Discussion

Given a promising activity against a wide range of resistant worm infections and its unique mode of action, it is worthwhile to evaluate the activity of emodepside against other helminth infections including STH and schistosomiasis. For the first time we thoroughly tested emodepside against a wide range of laboratory models for these diseases.

Both the larval and the adult nematode and schistosome stages were screened phenotypically in presence of emodepside over a time course of 72 hours followed by *in vivo* studies. As emodepside belongs to the group of cyclooctadepsipeptides that are known to be very active against different animal gastrointestinal nematodes and filarial parasites, good antinematicidal activity was expected [20, 30, 37–40]. The drug showed a high efficacy *in vitro* against all the nematode species and was highly effective against the two hookworms (*A. ceylanicum* and *N. americanus*) and the whipworm (*T. muris*). On the contrary, the effect of emodepside on schistosomes, remained only moderate.

We further investigated the efficacy of emodepside *in vivo* on rodents infected with *T. muris*, *A. ceylanicum* and *N. americanus.* Overall, the promising *in vitro* activity of emodepside was confirmed *in vivo*, where the drug demonstrated high worm burden reduction rates, even when administered orally as a low, single dose regimen.

These results were consistent with previous findings. The ED_50_ value obtained *in vivo* for *T. muris* (ED_50_ of 1.2 mg/kg) was very similar to the one reported by Kulke et al. [28]. Our study also confirms the good activity of emodepside *in vitro* against larval and especially adult stages of the nematodes *S. ratti* and *H. polygyrus* that was so far only described *in vivo* [29].

Emodepside performed much better *in vitro* and *in vivo* than albendazole, levamisole and pyrantel pamoate, the standard drugs used against STH infections tested in a previous study [36], where none of the standard drugs showed *in vitro* activity against adult *A. ceylanicum* (Table 3). Moreover, only a moderate *in vitro* efficacy against *T. muris* and *N. americanus* was reached by levamisole and pyrantel pamoate. In contrast, emodepside was very active *in vitro* against the larvae and adult worms of all three species. *In vivo*, albendazole was the only drug that performed as well as emodepside on *A. ceylanicum* infected hamsters (Table 4). While none of the three standard drugs cured mice harboring a *T. muris* infection, emodepside was fully active at a concentration of 75 mg/kg.

**Table 3:** Mean IC_50_ values (µg/ml) after 72 hours drug exposure on L3 and adult stages of *A. ceylanicum*, *N. americanus* and *T. muris* of emodepside compared to the ones of albendazole, levamisole and pyrantel pamoate

Species	Mean IC_50_ (µg/ml) after 72 hours of drug incubation			
	Emodepside	Albendazole^a^	Levamisole-HCl^a^	Pyrantel pamoate^a^
*T. muris* (L1) after 24 h	4.18			
*T. muris* (L3)	–	≥ 200	33.1	95.5
*T. muris* (adults)	0.022	≥ 200	16.5	34.1
*A. ceylanicum* (L3)	0.28	32.40	1.60	90.9
*A. ceylanicum* (adults)	0.0027	≥ 100	≥ 100	≥ 100
*N. americanus* (L3)	0.090	≥ 100	0.50	2.0
*N. americanus* (adults)	0.0024	≥ 100	13.40	7.6

^a^All values for this drug are taken from the study of Tritten et al. [36]

**Table 4:** *In vivo* dose response relationships of emodepside, albendazole, levamisole and pyrantel pamoate on *A. ceylanicum*, *N. americanus* and *T. muris*

Drug	Dose (mg/kg)	Worm expulsion rate (%)	Worm burden reduction (%)
*T. muris*			
Emodepside	75	100	100
	10	62.0	85.9
	2.5	60.9	69.6
	1.25	5.3	73.9
Albendazole^a^	600	49.4	20.2
Levamisole-HCl^a^	200	90.5	95.9
Pyrantel pamoate^a^	300	9.4	0
*A. ceylanicum*			
Emodepside	2.5	100	100
Albendazole^a^	1.25	70.5	87.8
	2.5	100	100
	5	100	100
Levamisole-HCl^a^	10	44.3	60.2
Pyrantel pamoate^a^	10	63.4	87.2
*N. americanus*			
Emodepside	10	100	100
	5	100	100
	2.5	87.5	100
	1.25	40.0	62.5
Albendazole^a^	10	100	100
	5	69.6	70.8

^a^All values for this drug are taken from the study of Tritten et al. [36]

Although emodepside was also very active *in vitro* on *H. polygyrus* L3 (with IC_50_ values below 1 µM), previous studies reported lower *in vivo* sensitivity of *H. polygyrus* larvae compared to the larval stages of other nematode species. This decreased activity against *H. polygyrus* larvae *in vivo* was explained by their presence burrowed deep into the gastro-intestinal tissues which was likely to protect them from the drug [29, 41]. Aiming at a formulation of emodepside active on all parasite stages, *in vivo* studies should preferably be performed at both early and late stages of infection. Hence future *in vivo* studies should evaluate the activity against the early developmental stages of the nematodes.

In our study, the *in vitro* activity of emodepside against the nematode species was higher in adult worms than in the larvae. Such difference in anthelmintic susceptibility between the early and the late developmental stages of the parasite was reported previously [42–44]. A differential expression of emodepside molecular target(s) between the parasites life-stages or differences in the permeability of the cuticle (or both) may account for it [42, 45]. Moreover, a similar trend was observed *in vivo* in other studies on *Nippostrongylus brasiliensis*, *S. ratti* and *H. polygyrus* [29]. However, for *S. mansoni*, we documented the opposite finding, with revealing lower IC_50_ values than the adults.

We observed in our *in vitro* studies that whereas the morphology of the parasites seemed not affected by the drug, often no motility or pharyngeal pumping movement could be detected. This observation corroborates the suggested mechanism of action of emodepside [30, 37, 46–49]. Although its exact mechanism of action is not fully understood yet, the drug is known to bind to two different targets of the neuromuscular junction, the evolutionary conserved calcium-activated potassium channel slowpoke 1 (SLO-1) and the latrophilin receptors LAT-1/LAT-2 [30, 37, 47, 50, 51]. In nematodes, the over activation of the SLO-1 receptors by emodepside is likely to induce a potassium efflux triggering a hyperpolarization of the neurons that results in a decreased synaptic transmission and muscle contraction, leading notably to a paralysis of the worm pharynx [37, 42, 47, 50, 52]. The specificity of emodepside towards the nematode channel subunits might account for its lack of efficacy against the trematodes [49].

The *in vitro* assay read-out methods varied among the different parasites and life-stages. While visual scoring of adult worms is generally straightforward for a trained operator, evaluating larval assays was more challenging, especially for *T. muris* and *S. ratti* larval assays. This led to a high variability between the different assays and explained a higher number of replicates reported compared to the other parasites. This finding urges the optimization and development of more accurate assessment methods.

## Conclusions

Our study confirms that emodepside represents a promising broad spectrum human anthelmintic drug candidate with intriguing activity against a wide range of nematodes. Since emodepside is already well characterized in veterinary medicine and undergoing clinical development for onchocerciasis, and the activity observed in this study against different nematodes was similar to previous findings on filarial worms [26] this will allow a significant shortcut developing this drug for human STH. A drug development plan should therefore be established to fill the missing gaps required so that emodepside will soon be available for the treatment of both filarial and STH infections.

## Additional files

**Additional file 1: Figure S1.** Mean IC_50_ overtime for the nematode L3.

**Additional file 2: Figure S2.** Mean IC_50_ overtime for the adult nematodes.

## Data Availability

The datasets supporting the conclusion of this article are included within the article and its additional files.

## Abbreviations

IC_50_: half maximal inhibitory concentration
ED_50_: median effective dose
STH: soil-transmitted helminthiases
WHO: World Health Organization
MDA: mass drug administration
HPLC: high-performance liquid chromatography
ACN: acetonitrile
TFA: trifluoroacetic acid
LC-MS: liquid chromatography-mass spectrometry
NMR: nuclear magnetic resonance
MS: mass spectrometry
DMSO: dimethyl sulfoxide
RPMI: Roswell Park Memorial Institute
L1: stage 1 larvae
L3: stage 3 larvae
iFCS: inactivated fetal calf serum
PBS: phosphate-buffered saline
NTS: newly transformed schistosomula
HBSS: Hanksʼ balanced salt solution
CO_2_: carbon dioxide
p.i.: post-infection
WBR: worm burden reduction
WER: worm expulsion rate
SLO-1: calcium-activated potassium channel slowpoke 1
LAT-1/LAT-2: latrophilin receptors
3 R: Replacement, Reduction, Refinement
