# Supplementary figures and images for: Evaluation of emodepside in laboratory models of human intestinal nematode and schistosome infections
Source: Parasit Vectors. 2019 May 14;12:226. doi: 10.1186/s13071-019-3476-x (PMC6515646; doi:10.1186/s13071-019-3476-x)

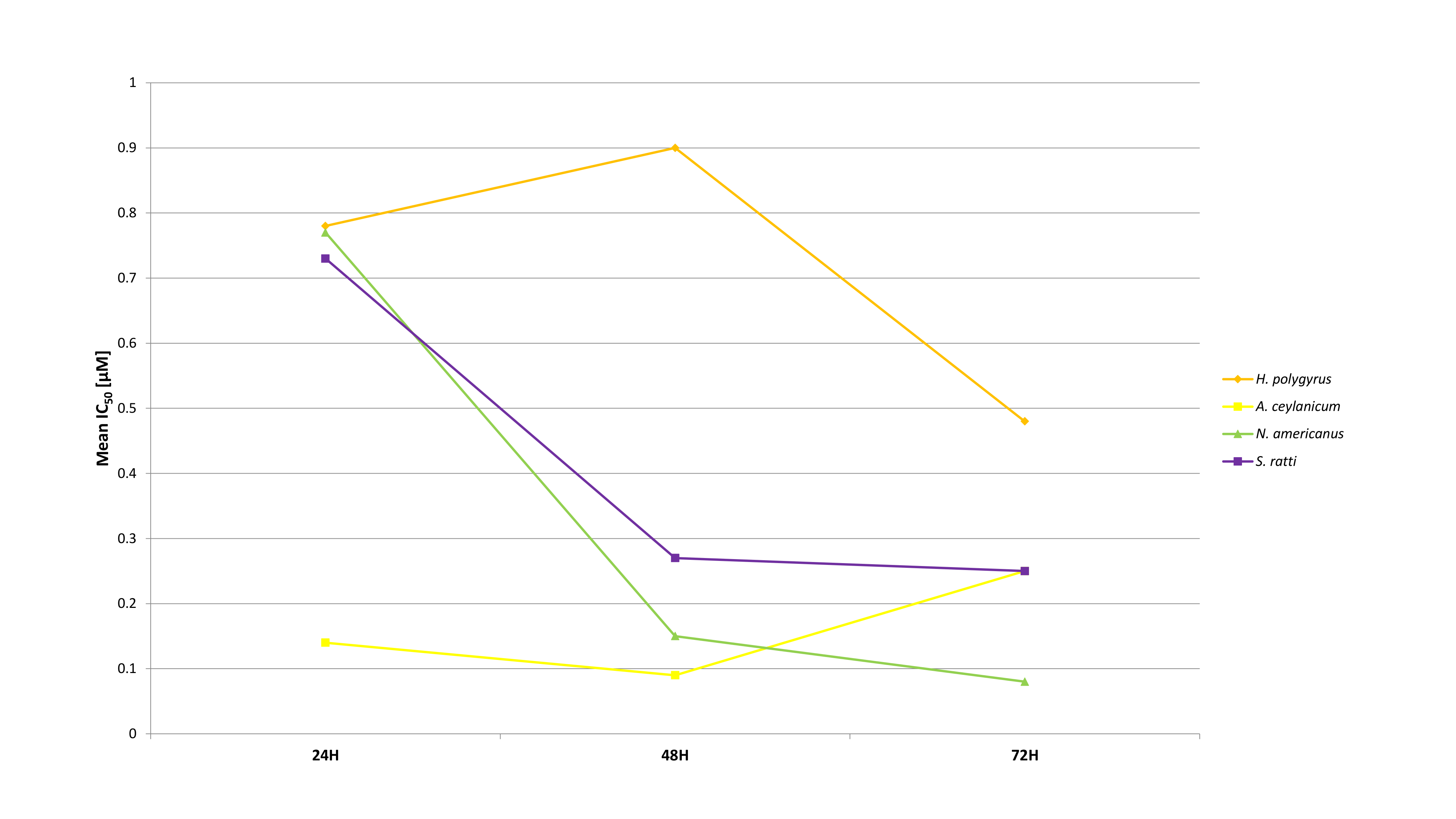

Supplement: Supplementary file 1 — Additional file 1: Figure S1. Mean IC50 overtime for the nematode L3. [file 13071_2019_3476_MOESM1_ESM.tif]

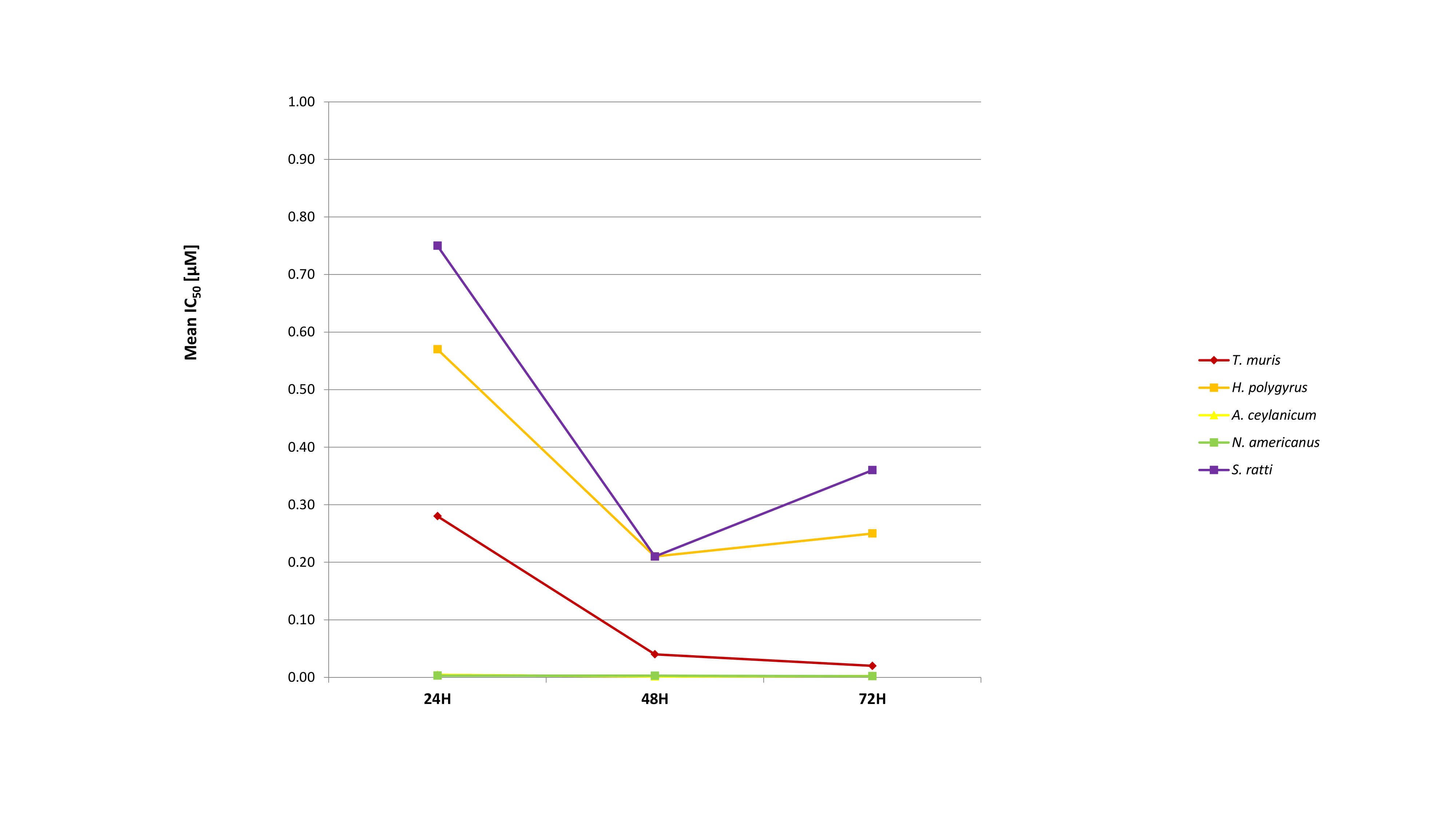

Supplement: Supplementary file 2 — Additional file 2: Figure S2. Mean IC50 overtime for the adult nematodes. [file 13071_2019_3476_MOESM2_ESM.tif]
